# Youth Perspectives on Barriers and Opportunities for the Development of a Peer Support Model to Promote Mental Health and Prevent Suicide

**DOI:** 10.1177/01939459221115695

**Published:** 2022-08-02

**Authors:** Jackie Libon, Jarlo Alganion, Carla Hilario

**Affiliations:** 1 Faculty of Nursing, University of Alberta, AB, Canada; 2 School of Nursing, Faculty of Health and Social Development, University of British Columbia, BC, Canada

**Keywords:** peer support, youth, suicide prevention, mental health, community, public health, community health nursing, community mental health services, rural health

## Abstract

Suicide prevention is a public health priority. The purpose of this study was to elicit and document the perspectives of youth (ages 15–24) on the development of a peer support model for mental health promotion and suicide prevention for youth in small communities in western Canada. A qualitative descriptive approach informed by a socioecological framework was used to conduct the study. Eleven youth participated in a series of three co-design workshops. Data collection was conducted remotely using Zoom. Data were analyzed using thematic analysis. The following three themes are identified: (a) contextual factors for youth; (b) community spaces and social media; and (c) apps and integrated care. These themes elucidated challenges faced by the youth, strategies for reducing barriers for youth who need support, and opportunities for enhancing youth mental health through responsive community-identified services. Overall, youth were supportive of the potential use of peer support to augment services in their communities.

Suicide is the second leading cause of death in Canadians aged 15–24 (Brådvik, 2018). Many among this cohort encounter extensive delays in seeking help or fail to seek help altogether, with those suffering from multiple mental illnesses among the most vulnerable (Brådvik, 2018). The United Nations defines youth as persons aged 15–24 years of age (n.d.). Youth suicide prevention strategies informed by evidence—and the input of those most at risk—are urgently needed across Canada. Nowhere is this truer than in rural communities, where suicide rates tend to be higher owing to reduced availability and accessibility of mental health services (Barry et al., 2021; Walker et al., 2009), and where mental illness is more heavily stigmatized (Pisani et al., 2012).

The COVID-19 pandemic has compounded these concerns through heightened anxiety and public health measures such as quarantine, social distancing, and self-isolation (Harris, 2020). While the pandemic has exacerbated mental health issues, there has also been an increase in capacity for telehealth, especially in mental health care, which has been helpful for reducing the disparities often encountered by rural communities (Cunningham et al., 2021). Hotlines, crisis centers, counseling services, public and physician education, school programs, and peer support have all been found to reduce suicide and attempted suicide among youth.

Peer support—namely that provided by individuals having a shared experience of a mental health condition (Shalaby & Agyapong, 2020)—stands out in particular through programs such as Sources of Strength (SOS), LifeSavers, and teen Mental Health First Aid (tMHFA). These programs have proven useful for developing coping skills and social connectedness (Wyman et al., 2010), enhancing self-esteem (Walker et al., 2009), and increasing mental health literacy (Hart et al., 2019). Young, rural Canadians nonetheless still face many challenges with regard to mental illness and suicidality, such as higher incidence and prevalence of suicide attempts and suicidal ideation (Barry et al., 2021), long wait times for treatment, and lack of youth and family perspectives in the programs currently available (Malla et al., 2018).

Peer support workers have been shown to mitigate mental health stigmas and mental health care costs while improving the quality of life for individuals (Bennett et al., 2015; Byrne et al., 2017). However, little is known regarding peer support and youth suicide prevention in rural settings. In this article, we report on a study engaging rural youth in conceptualizing a peer support model based on their experiences of mental health and service use and optimized for their unique community context (Simmons et al., 2018).

## Purpose

The purpose of this study was to elicit and document the perspectives of youth (ages 15–24 years) on the conceptualization and development of a peer support model for youth mental health promotion and suicide prevention in rural settings. In doing so, we sought to identify barriers and opportunities for improving youth mental health promotion and suicide prevention programming from the perspectives of youth, based on their lived experiences.

## Methods

We employed a qualitative descriptive approach to investigate the nuances of lived experiences, perspectives, and systems influencing youth mental health needs and access to services (Vaismoradi et al., 2013). To inform our approach, we drew on Bronfenbrenner’s (1977) socioecological framework to guide recruitment, data collection, analysis, and reporting of results. Bronfenbrenner’s (1977) socioecological framework of adolescent development situates youth at a nexus of various influences: microsystem, mesosystem, exosystem, and macrosystem. The microsystem comprises the immediate environment such as home, workplace, or schools; the mesosystem comprises the affiliations between the major systems; the exosystem comprises social structures or major institutions of society, including the government or health sector; and the macrosystem comprises the ideologies and belief systems pertaining to the other systems.

We employed co-design methods, described in more detail later in the data collection section below, to facilitate the engagement of youth participants and to elicit their perspectives in the conceptualization of a peer support model (Brown & Wyatt, 2010; Hackett et al., 2018). In keeping with provincial COVID-19 public health measures, we carried out all stages of the study remotely. As many mental health services were being offered remotely at the time (Government of Canada, 2021), we found this to be an acceptable method for data collection. Ethics approval was sought and obtained from the research team’s university research ethics board.

### Setting and Recruitment

The study was conducted in partnership between the study principal investigator and community-based organizations located in small, rural communities in western Canada with a population size of less than 20,000 and located approximately 50 kilometers from the nearest major metropolitan center. The community partners facilitated recruitment through their networks and email listservs. Respondents who met the inclusion criteria (i.e., community residents aged 15–24) received the study information package and a link to REDCap, a secure web application, for providing informed consent (Harris et al., 2009). Participants below the age of majority (those younger than 18 years) gave consent together with their parents or legal guardians. Through convenience and snowball sampling (Bhardwaj, 2019), we recruited 11 participants aged 15–24, the median age being 20. As this project was conducted as part of a community partnership, we followed the guidance of our community partners in planning our recruitment strategy. The lived experience of suicidality was not an inclusion factor as youth who have mental health concerns may have a higher risk for suicidality and could have valuable perspectives to contribute about mental health promotion and suicide prevention.

### Data Collection

**Figure 1.**
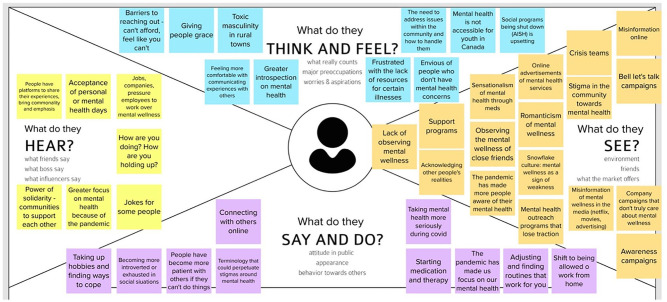
Empathy Mapping from Co-design Workshop #1.

In June and July 2021, the participants took part in three sequential participatory co-design workshops, each lasting 1.5 hours, using the Zoom teleconference platform. The workshops, intended to engage participants in conceiving a prospective peer support model for mental health promotion and suicide prevention in their community, were based on three stages of design thinking: Inspiration, Ideation, and Implementation (Brown & Wyatt, 2010). Session one (Inspiration) focused on opportunities and challenges related to mental health promotion and suicide prevention as identified by the youth participants. This first session included exercises (e.g., “Lightning Decision Jam” and empathy mapping) to elicit youth input on what they see, hear, say, think, and feel with regard to mental health and suicide (Figure 1). Session two (Ideation) focused on possible responses or solutions to the challenges identified. Participants engaged in exercises (e.g., “How Might We,” “analogies,” and “Creative 8s”) to facilitate the generation of ideas for incorporating peer support in their community (Supplemental Figure S1). Session three (Implementation) focused on conceptualizing possible strategies for advancing some of the ideas generated in session two into models that could be potentially implemented. Working in two groups, the participants narrowed down ideas from the previous session and discussed strategies for implementation in their community. The participants’ output, including their written ideas and illustrations, was collected together with our detailed field notes from each session which included (but were not limited to) observations from discussions, participant quotes, and key recurring themes. Field notes were recorded by a research team member present at each workshop.

### Data Analysis

We employed inductive and deductive thematic analysis to discern patterns in the data pertaining to mental health promotion, suicide prevention, service provision, and peer support (Braun & Clarke, 2006). Using NVivo© 12 qualitative software, we inductively developed a codebook through iterative engagement with the dataset and team consensus. Once these inductive codes were saturated and the data coded accordingly, we deductively applied the socioecological framework, drawing on Bronfenbrenner’s (1977) key concepts to inform the analysis of the themes. We assigned pseudonyms to all participants for analysis and project output.

### Rigor

We established rigor by building rapport with the participants, documenting their ideas with diligence, and corroborating our preliminary findings with them throughout the sessions. By remaining self-aware of our researcher roles, maintaining an audit trail, and capturing field notes throughout the process, we ensured our findings would be confirmable. Through every stage of the study, we took care to gauge the fitness of our methods to our research aims and remain transparent about our limitations to ensure our findings would be valid. We moreover tested our findings against the entire dataset to ensure their consistency and reliability by creating coding schemes independently and comparing our findings.

## Results

This article reports findings from the analysis of the field notes and participants’ outputs from the co-design workshops. The following three main themes are identified: (a) contextual barriers for youth; (b) community spaces and social media; and (c) apps and integrated care.

### Contextual Factors for Youth

Through the workshops, participants shared their day-to-day experiences and the challenges of living in rural communities. Their experiences were shaped by their immediate needs and intertwined with the context of their lives, including available services and support in their communities and the COVID-19 pandemic.

Structural barriers to care and support, such as long wait times, availability of programs, and lack of resources and funding, were prominent in the data and were largely attributed to the rural context of their communities. Participants characterized financial inequities as a major issue; the expenses associated with mental health services left Marie feeling “freaked out,” (age 21) and another participant noted being “afraid to reach out for help.” Participants were further concerned about the cost and availability of transportation to and from in-person services and the role of the government in funding and sustaining programs. “Waiting for the government to provide support for mental health is like waiting for rain in a drought,” said Avery (age 23). From a socioecological standpoint, these factors were all indicative of exosystemic or societal influences, both proximal and distal, on participants’ mental health and health care experiences.

A second, broad aspect of participants’ immediate lived experience was the COVID-19 pandemic, which significantly shaped their everyday lives and their mental health. Participants widely agreed that social withdrawal, difficulty communicating with others, and struggling to cope with stressors had all become major issues in their lives since the COVID-19 outbreak. At the same time, participants perceived a greater public discourse and de-stigmatization of discussions around mental health, suicide, and self-care as the lockdowns and other public health restrictions wore on. The pandemic brought about new mindfulness of day-to-day personal mental health.

In the words of Spencer (age 24), “When [youth] have to stay home, they can realize that there are some people in [their] lives that can be toxic or demanding. Separation from others helps to draw attention to the fact that personal mental health needs attention.” COVID-19 brought about an earnest desire among the youth to take stock of their mental wellness on a regular basis.

### Community Spaces and Social Media

The participants’ comments and output illustrated that communities play a significant role in reducing barriers to support and facilitating a sense of belonging, which was viewed as critical to mental health and suicide prevention. Through community support and programming and the selective use of social media, participants reported experiencing rewarding feelings of belonging and connectedness. This sense of community could be regarded as exosystemic and macrosystemic within the socioecological framework.

As Haven described (age 21), mental health promotion requires “whole communities working together, mingling together, being friends with each other, and having spaces where someone who struggles with mental illness or addiction might feel more comfortable.” The participants found that connecting with others in the community was an important aspect of caring for their mental health and should be included in mental health initiatives. While gaps in mental health community resources and crisis prevention services were foremost among participants’ concerns, they also spoke highly of existing mental health programs that create an authentic sense of belonging. In addition, valued spaces were also seen as those that countered mental illness stigma and fostered a sense of inclusion for youth living with mental illness.

Social media was viewed as another important venue for promoting community and mental health. Participants expressed that online platforms such as Facebook, Discord, and Reddit had the potential to improve psychosocial wellness by providing a space to share experiences and find connections. In the words of Spencer (age 24), “the internet gives a voice to a lot of people that wouldn’t otherwise have a platform to speak, which creates the opportunity to share experiences and create solidarity.” The participants found social media to be a great place to share their experiences and connect with others to enhance their mental health.

The participants perceived additional benefits to social media such as promoting public awareness about mental health and mental illness, disseminating information about coping strategies, and even linking to tools for mental health self-assessment. For example, Stormy (age 23) described watching a TikTok video wherein the subject shared their experience of living with the same, unique symptoms that Stormy was experiencing. Stormy credited this experience for providing the impetus to seek professional intervention for attention deficit hyperactivity disorder (ADHD). In Stormy’s words, “It’d be really cool to have a social media type platform where everyone could share their experiences [to] help each other figure out what they might be dealing with and how others deal with it already.” Many of the youth agreed with Stormy, noting the importance of connecting with others through shared experiences.

### Apps and Integrated Care

In the final co-design workshop, the participants developed their ideas for responsive youth mental health care and suicide prevention programming in their community by conceptualizing prototypes for a peer support model. Their ideas took the form of two conceptual prototypes for developing initiatives: a mental health app and an integrated care model placing all mental health services in one location. The underlying principles—accessibility and centralization—could be considered exosystemic insofar as they emphasized the roles of policymakers, social services, and mass media.

The conceptual prototype of a mental health app was seen as particularly responsive to the conditions created by the COVID-19 pandemic. The pandemic, and the resulting closure of some in-person services, diverted many of the participants to seek mental health care online. As residents of a rural community, they were further drawn by the appeal of instant access to mental health services unburdened by long distances or wait times. From this line of discussion emerged the consensus that a mental health app, facilitating virtual peer support, would benefit their community through COVID-19 lockdowns and beyond. The participants drafted details such as a welcome page, privacy safeguards, and a system for booking virtual or in-person appointments. The app would be developed and implemented by a diverse group of local stakeholders including health professionals, businesses, parents, youth, community support workers, teachers, and social workers to create an “environment where you meet and know someone before you treat them, to establish trust first,” as Spencer (age 24) put it. The participants agreed with Spencer as establishing trust appeared to be a vital first step to mental health treatment.

Most importantly, the app would be created by and for the members of a specific community, thereby representing a meaningful, mutual investment in local public health. From this prototype initiative for peer support, participants foresaw outcomes such as long-term peer connections, increased awareness of local mental health support, and a sense of community as fostering overall well-being. The youth viewed a mental health app as one potential model to access peer support, streamline the appointment booking process, and establish therapeutic relationships.

The second conceptual prototype initiative was a one-stop shop or integrated mental health care model, placing all programs under one roof, including peer support, ideally within a community center. Stormy (age 23) likened this model to “an amusement park, with all the rides in one place”—a well-planned, collaborative environment allowing for seamless and comfortable service provision. Among the embedded services would be peer support for at-risk youth. In Avery’s (age 23) words, it would provide “a shared space prior to crisis.” Several participants mentioned this shared space as having the possibility to find resources and support in one another as well as motivation and desire for self-care, thereby ensuring that prevention and early intervention would receive due emphasis.

Overall, the participants agreed on the importance of having a variety of professionals and peers in one place as they can all impact one’s mental health. They enjoyed the idea of a centralized care model that recognizes how each individual may require different support due to differing experiences. They discussed the significance of knowing others are there to support you, even when you do not ask for it, in enhancing relationships, building trust, and ultimately improving one’s mental health.

## Discussion

This study was designed to engage youth in a discussion of peer support for mental health promotion and suicide prevention in their community. This study brought to light systemic barriers to accessibility, affordability, and availability of mental health services and perspectives on developing a peer support model for this cohort. In our application of Bronfenbrenner’s (1977) socioecological framework, we found that participants’ perspectives on community spaces and social media could be situated in the exosystem and macrosystem, which include connecting with others via support programs or online platforms. This socioecological viewpoint has value in locating the needs and vulnerabilities particular to rural youth, vis-à-vis suicide prevention and mental health, in a societal and community context.

Mobile applications have received increasing attention in the youth mental health literature. Seko et al. (2014) found that mobile applications for youth mental health are beneficial to the extent they can promote emotional self-awareness, adherence to treatment, and equitable access to resources; however, such technology may also present concerns with confidentiality, technicality, and cost. Any mobile app initiative to streamline peer support services would need to address these issues prior to implementation.

Similarly, there is growing research on the use of integrated care models in providing services for youth. Previous research has found that integrated youth mental health care models can facilitate early prevention, community engagement, partnerships, and collaboration (Settipani et al., 2019). McGorry et al. further discuss how integrated care models are better suited to address the mental health needs of 21st-century youth compared to traditional models, when used to engage youth in the design and implementation within a community, in promoting sustained long-term care and youth empowerment (2022). Thus, integrated care models hold promise for addressing the unique needs of youth and should be considered when developing a peer support model for mental health and suicide prevention efforts. However, it is also important to consider that rural areas often contain higher stigmatization of mental health and lack adequate mental health providers, which could be a challenge to implement this model (Goodcase et al., 2021).

It is noteworthy that social media was prominent in much of the participants’ discussion. Research has shown social media can increase youth self-esteem, social connectedness, and confidence, leading to healthy identity development (Uhls, 2017). TikTok, in one instance, has been used to create peer-to-peer communities for support and social connectivity, targeting youth during the pandemic (Literat, 2021). Such platforms therefore show considerable potential for spreading awareness of mental health and treatment options among young people living in rural settings and could be linked to an app for peer support for youth. This observation nonetheless comes with a major caveat, namely the well-documented risks for cyberbullying and poor health outcomes associated with excessive screen time (Uhls, 2017).

All of the youth in this study struggled with daily life owing to the COVID-19 pandemic, yet this set of challenges was not without positive aspects. The pandemic provided an occasion to take stock of their mental health. For participants, isolation meant fewer distractions and more opportunities to reflect on psychosocial wellness, connect online with others, and achieve a better work-life balance. While the deficits of COVID-19 on youth mental health have been extensively documented (Courtney et al., 2020; Liang et al., 2020; Power et al., 2020), the benefits that participants of this study derived suggest that youth can also positively respond to adverse circumstances such as a global pandemic. This is a finding that warrants further investigation. In such dire, global public health circumstances, teachable coping strategies may be gleaned from the adaptability of young people. As this study was aimed at conceptualizing a peer support model for youth, as informed by their perspectives on contextual factors in their communities, future research could focus on determining what a peer support model would look like more concretely.

### Limitations and Strengths

Our data collection modality resulted in some limitations. Conducting the co-design workshops remotely—unavoidable during the COVID-19 public health restrictions—may have prevented some youth from taking part, in particular those whose socioeconomic circumstances did not allow easy access to a private and/or stable internet connection. The sample size was smaller than optimal and demographic data were limited to place of residence and age. Additional information, such as gender and ethnicity, might have been beneficial for contextualizing the findings and providing additional layers of interpretation. Notwithstanding our modest sample size, the three co-design workshops provided rich qualitative data, sufficient to achieve saturation. The methodology furthermore empowered participants as partners in interpreting and organizing the data and creating project output.

### Practice Implications

This study elicited youth perspectives on a conceptual peer support model for mental health promotion and suicide prevention, including potential initiatives to facilitate access, such as a mental health peer support app and an integrated care model with peer support. Our findings add to the body of knowledge around youth mental health and suicide prevention, especially with regard to peer support programming. In addition, this study advances the evidence base for youth mental health service provision and suicide prevention in rural settings. The project output could inform community programming and policies to support the development and delivery of youth mental health services and community-based suicide prevention.

## Conclusion

Our findings indicate rural youth welcome peer support as a mental health intervention, and it may be an effective suicide prevention model for this cohort. The barriers—and potential assets—to accessing peer support are all exosystemic and macrosystemic, meaning it is necessary to draw on the collective power of policymakers, health care and other service providers, media, and community leaders to bring about improvements in service delivery, public awareness, and attitudes toward mental illness and suicide. Remarkably, the COVID-19 pandemic may already have helped transform public discourse in this regard; certainly, it has inspired youth to be more aware and proactive with regard to their mental well-being. In the current climate of profound stress on global public health and service provision, this may represent a hopeful trend.

## Supplemental Material

sj-pdf-1-wjn-10.1177_01939459221115695 – Supplemental material for Youth Perspectives on Barriers and Opportunities for the Development of a Peer Support Model to Promote Mental Health and Prevent Suicide Click here for additional data file. Supplemental material, sj-pdf-1-wjn-10.1177_01939459221115695 for Youth Perspectives on Barriers and Opportunities for the Development of a Peer Support Model to Promote Mental Health and Prevent Suicide by Jackie Libon, Jarlo Alganion and Carla Hilario in Western Journal of Nursing Research
