# Supplementary figures and images for: Youth Perspectives on Barriers and Opportunities for the Development of a Peer Support Model to Promote Mental Health and Prevent Suicide
Source: West J Nurs Res. 2022 Aug 2;45(3):208–14. doi: 10.1177/01939459221115695 (PMC9902963; doi:10.1177/01939459221115695)

## Examples of participant-generated ideas from the Creative 8's exercise in Co-Design

### Workshop #2

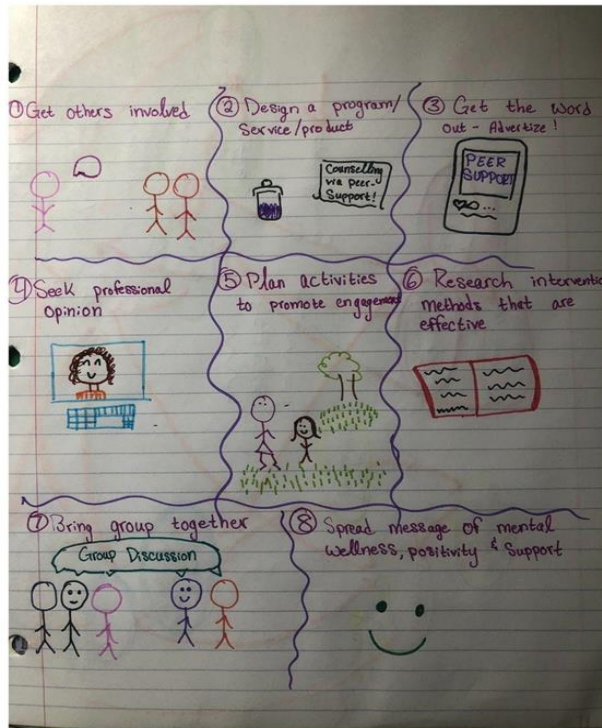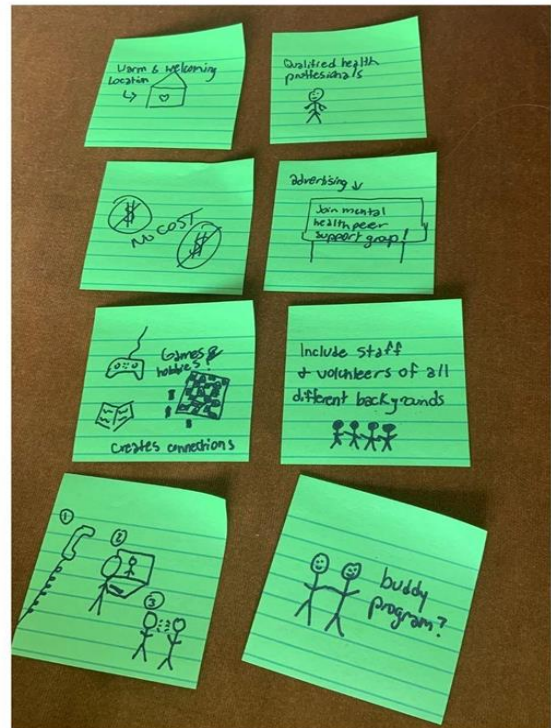

Supplement: sj-pdf-1-wjn-10.1177_01939459221115695 – Supplemental material for Youth Perspectives on Barriers and Opportunities for the Development of a Peer Support Model to Promote Mental Health and Prevent Suicide [file sj-pdf-1-wjn-10.1177_01939459221115695.pdf]
